# Alternative Splicing of Opioid Receptor Genes Shows a Conserved Pattern for 6TM Receptor Variants

**DOI:** 10.1007/s10571-020-00971-7

**Published:** 2020-10-03

**Authors:** Marjo Piltonen, Andrey Krokhotin, Marc Parisien, Pierre Bérubé, Haig Djambazian, Rob Sladek, Nikolay V. Dokholyan, Svetlana A. Shabalina, Luda Diatchenko

**Affiliations:** 1 School of Dentistry, McGill University, Genome Building, Room 2201, 740 Dr. Penfield Avenue, Montreal, Quebec H3A 0G1 Canada; 2 Department of Anesthesia, School of Medicine, McGill University, Genome Building, Room 2201, 740 Dr. Penfield Avenue, Montreal, Quebec H3A 0G1 Canada; 3 Alan Edwards Centre for Research on Pain, McGill University, Genome Building, Room 2201, 740 Dr. Penfield Avenue, Montreal, Quebec H3A 0G1 Canada; 4 Departments of Pathology, Genetics and Developmental Biology, Stanford Medical School, Howard Hughes Medical Institute, Palo Alto, CA 94305 USA; 5 Departments of Human Genetics and Medicine, Faculty of Medicine, McGill University, Montreal, Quebec H3A 0G1 Canada; 6 McGill University and Génome Québec Innovation Centre, Montreal, Quebec H3A 0G1 Canada; 7 Departments of Pharmacology, and Biochemistry & Molecular Biology, Penn State College of Medicine, Hershey, PA 17033-0850 USA; 8 Departments of Chemistry, and Biomedical Engineering, Penn State, University Park, PA 16802 USA; 9 National Center for Biotechnology Information, National Library of Medicine, National Institutes of Health, Building 38A, Room S604, 8600 Rockville Pike MSC 3830, Bethesda, MD 20894-6075 USA

**Keywords:** Opioid receptor, Alternative splicing, Evolutionary conservation, Gene enrichment

## Abstract

The opioid receptor (OPR) family comprises the mu-, delta-, and kappa-opioid, and nociceptin receptors that belong to the superfamily of 7-transmembrane spanning G protein-coupled receptors (GPCRs). The mu-opioid receptor is the main target for clinically used opioid analgesics, and its biology has been extensively studied. The N-terminally truncated 6TM receptors isoform produced through alternative splicing of the *OPRM1* gene displays unique signaling and analgesic properties, but it is unclear if other OPRs have the same ability. In this study, we have built a comprehensive map of alternative splicing events that produce 6TM receptor variants in all the OPRs and demonstrated their evolutionary conservation. We then obtained evidence for their translation through ribosomal footprint analysis. We discovered that N-terminally truncated 6TM GPCRs are rare in the human genome and OPRs are overrepresented in this group. Finally, we also observed a significant enrichment of 6TM GPCR genes among genes associated with pain, psychiatric disorders, and addiction. Understanding the biology of 6TM receptors and leveraging this knowledge for drug development should pave the way for novel therapies.

**Electronic supplementary material:**

The online version of this article (10.1007/s10571-020-00971-7) contains supplementary material, which is available to authorized users.

## Introduction

G protein-coupled receptors (GPCRs) are a superfamily of complex signaling proteins with over 800 members. Canonical GPCRs have seven helical transmembrane domains (TM) connected with intra- and extracellular loops, an extracellular N-terminus, and an intracellular C-terminus (Supplementary Fig. 1). GPCRs respond to a plethora of stimuli from photons to neurotransmitters and hormones, conveying their message on intracellular effector proteins. They are highly relevant therapeutic targets as approximately 1/3 of small-molecule drugs on the market act on GPCRs (Santos et al. 2017).

While differential RNA processing affects many GPCR transcripts, not much is known about the roles of truncated GPCR variants. In general, truncated GPCRs are created by alternative splicing, usage of an alternative transcription start site, or alternative polyadenylation site in the GPCR gene, typically in a very context-dependent manner (e.g., tissue-specific expression) (Wise 2012). We are only beginning to understand the diversity they create in the signaling landscape of a particular receptor.

We were interested in truncated receptor isoforms in OPR family, the mu-, delta-, kappa-opioid, and the nociceptin receptors (MOP, DOP, KOP, and NOP/ORL1). MOP is the main target for opioid analgesics and therefore clinically important. The OPRs display a notable range of the functional complexity of GPCRs: oligomerization, allosteric modulation, constitutive activity, complex trafficking and recycling, biased agonism and signaling from intracellular compartments (Al-Hasani and Bruchas 2011; Geppetti et al. 2015). The MOP is also well represented in a rather neglected field of GPCRs: the contribution of truncated GPCRs to the functional landscape. It has been shown that alternative splicing of the *OPRM1* gene coding for MOP produces mRNA transcripts that are translated to (i) 7TM receptors with amino acid variation in both the C- and N-termini, (ii) N-terminally truncated 6TM receptors, and (iii) 1TM fragments of the first transmembrane domain (Pasternak and Pan 2013; Convertino et al. 2015a). Previous studies have shown that 6TM MOPs are functional receptors with distinct excitatory cellular effects (Gris et al. 2010; Samoshkin et al. 2015; Convertino et al. 2015b) that provide potential targets for efficient analgesics, and play a prominent role in opioid tolerance, dependence, and opioid-induced hyperalgesia (Marrone et al. 2017). Despite their potential to affect OPR signaling and function, it is not known whether 6TM isoforms are common among other OPRs and generally among GPCRs.

All OPRs are coded by single multi-exonic genes, namely *OPRD1, OPRM1, OPRK1, and OPRL1*. They apparently evolved from a single ancestral OPR gene through two rounds of genome duplication, and the four receptor types arose already very early in the vertebrate evolution (Stevens et al. 2007; Dreborg et al. 2008). Their receptor topologies are conserved, and alternative transcripts exist for all of the OPR genes (as seen in Ensembl, NCBI Gene, and UCSC Browser). Genome sequence analysis shows that the human and mouse *OPRM1* genes share a similar structure, and that most human *OPRM1* exons have corresponding exons in the mouse at varying levels of divergence (Shabalina et al. 2009). In this regard, *OPRD1* was different: alternative exons in mouse and human do not have corresponding orthologs in the other species and are conserved only in closely related species (Piltonen et al. 2019). However, similar genetic organization allows for all OPR genes to code for 1TM, 6TM, and 7TM receptors. The contribution of the truncated receptors to functional opioid effects is virtually an unexplored area: the 6TM variants are particularly intriguing, as they have different and unique signaling capabilities even though they contain most of the functional domains of the receptor and the ligand-binding pocket is also largely unaffected (Gris et al. 2010; Samoshkin et al. 2015; Convertino et al. 2015b).

The aim of this study was to comprehensively study the alternative splicing in the 5′ regions of OPR genes to identify N-terminally truncated variants, and to explore their evolutionary conservation pattern. We also performed an analysis of gene databases and literature to explore if N-terminally truncated 6TM receptor variants are a common phenomenon of GPCR genes in general.

## Methods

### 5′RACE of Opioid Receptor Genes

#### RNA Extraction

Cell culture for human BE(2)-C neuroblastoma cells was performed as described previously (Piltonen et al. 2019). RNA was extracted with the guanidinium thiocyanate-phenol–chloroform procedure. The cells were stimulated with 100 nM [D-Ala2]-Deltorphin II for 1 h since this treatment induced an upregulation of MOR-1, MOR-1K, and DOR-1 in a pilot experiment (data not included). After the treatment, cells were collected in 1 ml of Qiazol and frozen at − 84 °C until processed. The samples were thawed and 200 µl of chloroform was added to each tube and mixed well. The samples were centrifuged at 4 °C, 12,000×*g* for 15 min to separate phases. The aqueous phase was gently transferred to a clean tube and mixed with 500 µl of isopropanol, incubated for 10 min at room temperature and finally centrifuged again to precipitate RNA. The RNA-pellet was resuspended and washed with 1 ml of 75% EtOH and re-pelleted at 4 °C, 7,500 g for 5 min. The pellet was air-dried and dissolved in 30 µl of RNAse-free, DEPC-treated water and heated for 10 min at 60 °C.

#### 5′RACE

Rapid amplification of cDNA ends (RACE) was employed to discover unknown exons or other splicing events occurring upstream of exon 2 in all OPRs, since the emphasis of the study was to discover transcripts coding for N-terminally truncated 6TM receptor isoforms. *OPRM1* was also primed from exon 13, which is known to be included in transcripts producing 6TM MOP. RACE-ready cDNA was created with SMARTer RACE kit (Clontech, USA), as instructed by the manufacturer from BE(2)-C or human brain total RNA (Clontech, USA). PCR with the universal forward primer from the kit and gene-specific reverse primers in exon 2 of each OPR gene (also exon 13 for *OPRM1*) was performed, followed by nested PCR for better specificity and yield of amplicons, and to ligate adaptor sequences for further barcoding of the samples. Each reaction was electrophoresed on a 1.2% agarose gel to verify the existence of PCR products, after which the samples were processed for sequencing with PacBio RSII and aligned with human genome hg19 (in collaboration with Genome Quebec, Canada). PacBio sequencing was performed as described in Piltonen et al. (2019).

Prediction of transmembrane helices was done with both TMHMM Server v.2 and TMpred (TMbase 25) to classify all OPR transcripts as sources of 7TM, 6TM, or 1TM receptor variants.

### Estimation of Evolutionary Rates and Selection Pressure

Human-macaque orthologous exon pairs and their coordinates were downloaded from the UCSC database or found by using the BLAT search procedure. The exon alignments were generated using the OWEN alignment tool (Ogurtsov et al. 2002) with the following criteria: a *P* value < 0.001 for each hit and sequence bounded at the 3′ or 5′ ends by exons aligning through > 80% of their length. Alignment of the CDS nucleotide sequences was guided by amino acid sequence alignment. Human-macaque exon alignments were generated for all four OPR gene loci.

The rates of divergence for exons with 5′ UTRs or 3′ UTRs were calculated using Kimura's two parameter model. For each exon, multiple alignments of 100 vertebrate species were retrieved, along with a 100-nt extension on both ends from the UCSC Genome Browser (https://genome.ucsc.edu). The conservation score of each exon was derived by averaging PhastCons scores (“Conservation Track” in the UCSC Genome Browser) which were assigned using a hidden Markov model-based method that estimates the probability that each nucleotide belongs to a conserved element based on the multiple alignment. PhastCons considers the phylogeny by which several species are related and uses statistical models of nucleotide substitution that allow for multiple substitutions per site and for unequal rates of substitution between different pairs of bases. Simply, the PhastCons score is the probability of negative selection, and the values range from 0 to 1.

### Ribosomal Profiling

The GWIPS-Viz database [Genome Wide Information on Protein Synthesis through the visualization of ribosome profiling data (Michel et al. 2014)] was utilized to find evidence of translation of transcripts coding for 6TM OPRs. GWIPS-viz browser is based on the UCSC Genome Browser (Genome Informatics Group, Center for Biomolecular Science and Engineering, University of California, Santa Cruz) and provides online tools for the interpretation of protein expression data obtained with ribosome profiling technique. We collected data for OPRs from both hg19 and hg38.

### Bioinformatic Analysis of the UNIPROT Database for 5-6TM GPCRs

We searched for GPCR splice isoforms in the UNIPROT database (The UniProt 2017). We first analyzed all primary protein isoforms in UniProtKB/Swiss-Prot (which contains manually curated UNIPROT entries), and identified all protein isoforms bearing 7-transmembrane (TM) helices that were members of the GPCR superfamily. Using annotations from Swiss-Prot we identified positions and sequences of all transmembrane domain for selected GPCRs. Next, we tested all GPCR splice isoforms from the UniProtKB/Swiss-Prot and UniProt/TrEMBL (which contains automatically annotated UNIPROT entries) for the presence of TM helices as identified based on comparison with corresponding primary isoform. All isoforms marked as Fragments in TrEMBL were excluded from the analysis. We selected isoforms that have at least 4 TM helices and miss the first or the seventh TM helices. If TMs were replaced with an alternative sequence, we checked it for potential to form TM helices using TMHMM Server v2.0 (Krogh et al. 2001) and Phobius (McWilliam et al. 2013).

### GPCR Enrichment Analysis

The enrichment of GPCR genes and N-terminally truncated 6TM GPCR genes among genes implicated in pain, psychiatric disorders, or addiction was evaluated by comparing the probability of a gene being both N-terminally truncated 6TM gene and a “disorder gene” [prob(GPCR ∩ Disorder)] to the individual probabilities of a gene being either a (6TM)GPCR [prob(GPCR)] or a disorder gene [prob(Disorder)]. The formula used is prob(GPCR ∩ Disorder)/[prob(GPCR)*prob(Disorder)]. The analyses included 824 GPCR genes (data from Uniprot) and 800 pain genes (Parisien et al. 2019). We also included 1383 genes (a subset of genes under the classifications of Schizophrenia spectrum and other psychotic disorders, Depressive disorders, and Bipolar disorders and related disorders) for psychiatric disorders genes from PsyGeNET (Gutierrez-Sacristan et al. 2015), and 383 addiction-related genes (Li et al. 2008). The lists of genes belonging to each category can be found in Supplementary Table 2. The total number of human genes at the time of this work was estimated at 19,020. The statistical significance of the enrichment was assessed using a binomial test comparing the expected frequency of (6TM)GPCR genes in the human genome with the observed frequency of those within the disorder genes.

## Results

### *OPRM1* Architecture Expands with Novel Cassette Exons and Alternative Splice Acceptor/Donor Sites

**Fig. 1:**
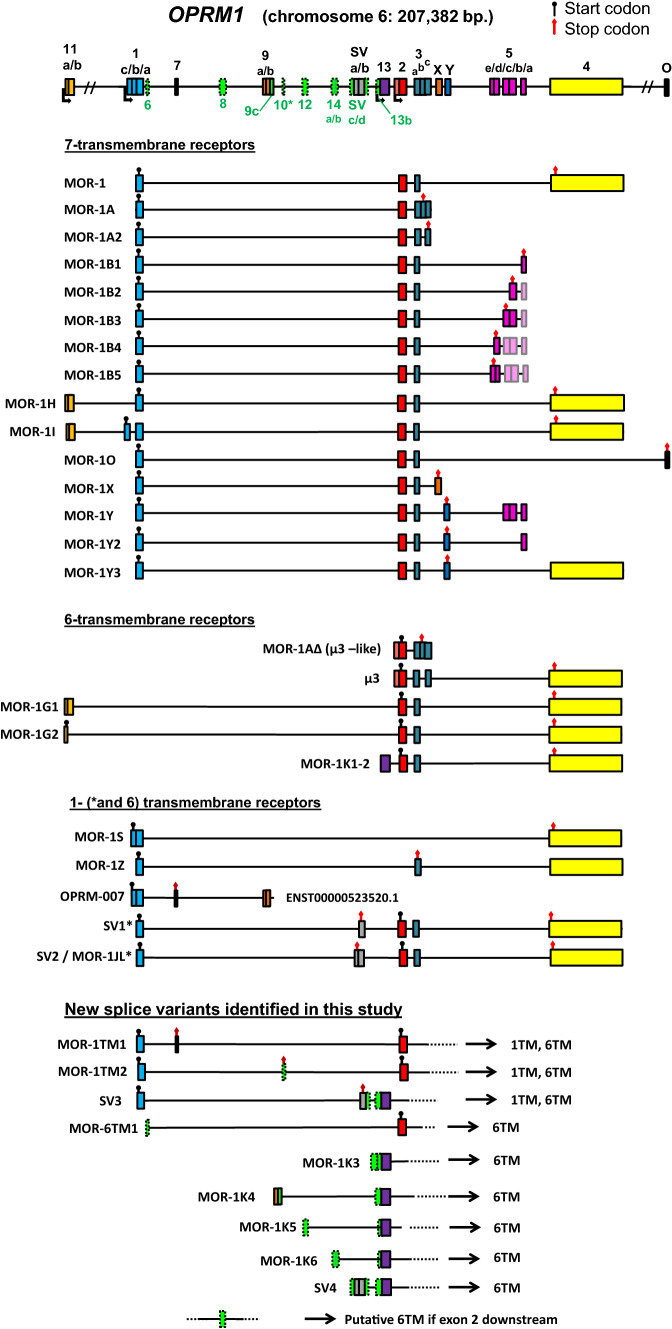
Human *OPRM1* gene structure and transcripts. Known exons and transcripts were identified using the UCSC browser and Ensembl. New exons discovered in this study are marked as green dashed-line boxes and named below the gene. Previously known exons are marked with solidly lined boxes filled with various colors for easy identification in the transcripts. Known transcription start sites are marked with an angled arrow; known or putative start codons are marked with a black circle, and stop codons are marked with a red diamond. An asterisk marks a new cassette exon that is putatively coding

Our experimental approach of 5′RACE PCR followed by deep sequencing yielded multiple transcripts with novel structures for *OPRM1* (Fig. 1, green and/or dashed boxes for previously unreported exonic structures). Since we focused on N-terminus variants of *OPRM1*, we initiated our RACE amplicons from exon 2. The vast majority of PCR products corresponded to a combination of exons 1 and 2, as expected, because they are found in all 7TM receptor isoforms (Table 1) and they represent the majority of *OPRM1* isoforms (Xu et al. 2014). Also MOR-1K (exons 13 + 2) and µ3 (5′ extension of exon 2 + exon 2) were seen frequently. In addition to the expected transcripts, we identified six cassette exons (Fig. 1. top trace, exons 6, 7, 8, 10, 12) between exons 1 and 2. Also, previously reported exons 7 and 9ab were seen in a different configuration (MOR-1TM1 and MOR-1K4, respectively) than previously reported (OPRM-007; ENST00000523520.1). Apart from new cassette exons, we also observed alternative splice acceptor sites upstream of 5′ end of exon SVa (called SVc) and exon 13 (exon 13b), and alternative splice donor sites beyond 3′ends of SVb (SVd) and exon 9 (exon 9c). However, these alternative splicing events are not located in the CDS regions. The existence of all exons was verified by RT-PCR (results not included), and in some cases we observed variability in the exonic junctions, suggesting the existence of additional splice acceptor and donor sites. Their detailed analysis, however, will be a matter of a separate study, as we only sought to verify the exons discovered by 5′RACE. Schematic representations of all novel transcripts are shown in Fig. 1, as they were identified in 5′RACE.

**Table 1:** Numbers of sequencing reads for specific exons or exonic junctions corresponding to the transcripts identified by the 5′RACE

OPRM1 Transcript(s)	OPRM1 Exons	Receptor type	# of reads brain	# reads BE(2)-C
All 7TM excl. MOR-1H, 1I (exon 11 zero reads)	1-2a	7TM	2481	1778
MOR-1AΔ, µ3	2ab	6TM	12	17
MOR-1K2	13-2a	6TM	33	81
MOR-1TM1	7-2a	1TM, 6TM	0	2
MOR-1TM2	10-2a	1TM, 6TM	6	6
SV3	1-SVa/b/c/d-13b	1TM, 6TM	0	72
MOR-6TM1	6-2a	6TM	18	0
MOR-1K3	13-13b only	6TM	0	59
MOR-1K4	9-13b	6TM	0	75
MOR-1K5	12-13b	6TM	15	0
MOR-1K6	14-13b	6TM	61	126
SV4	SVa/b/c/d-13b	6TM	39	89

OPRD1 Transcript(s)	OPRD1 Exons	Receptor type	# of reads brain	# reads BE(2)-C
DOR-1	1-2	7TM	2778	1233
DOR-1B	Exon 6	7TM	0	13
DOR-1C	Exon 4	1TM, 6TM	17	0
DOR-1D	Exon 5	1TM, 6TM	0	6
DOR-1E	2b	6TM	49	349

OPRK1 Transcript(s)	OPRK1 Exons	Receptor type	# of reads brain	# reads BE(2)-C
KOR-TV1, TV3, TV6a, TV6b	1-2	7TM	3961	Not expressed
KOR-TV3	1ab	7TM	24	
KOR-TV2	5-2	1TM, 6TM	53	
KOR-TV5	7-4a	4TM	121	
KOR-TV6a	4ba	7TM	333	
KOR-TV6b	7-4a	7TM	121	
KOR-TV7	8-2	6TM	265	

OPRL1 Transcript(s)	OPRL1 Exons	Receptor type	# of reads brain	# reads BE(2)-C
NOP-TV1	4-7	7TM	330	852
NOP-TV2	4-1	7TM	174	208
NOP-4b	1c-1a	7TM	83	6
NOP-TV5b	7a-1ab	6TM	2	0
NOP-TV6 (NOP-TV3 zero reads)	6-1a	7TM	24	1
NOP-TV7a	4-8a	7TM	20	0
NOP-TV7b, NOP-TV8a	4-8b	7TM	15	0
NOP-TV8a	8a-7b	7TM	3	204
NOP-TV9a, NOP-TV13c	7ab (no ex8)	7TM	296	107
NOP-TV9b	7c	7TM	406	293
NOP-TV10	1-9-2	1TM, 6TM	5	0
NOP-TV11	2ab	6TM	298	1089
NOP-TV12	LKAAEAR1 intron-7a	7TM	0	31
NOP-TV13a	LKAAEAR1 intron-6	7TM	0	24
NOP-TV13b	LKAAEAR1 intron-6-7a	7TM	0	16
NOP-TV13c	LKAAEAR1 intron-6-7b	7TM	0	16
NOP-TV14ab (NOP-TV3 zero reads)	5	7TM	13	0

Exons 7 and 10 disrupt the open reading frame starting from exon 1 with premature stop codons (PTCs) in transcripts MOR-1TM1 and MOR-1TM2. SV3, which resembles SV2/MOR-1JL, also introduces a PTC. These transcripts may be candidates for nonsense-mediated decay (NMD), but also hold the potential to code for a 1TM receptor fragment terminating in the PTC and a putative 6TM receptor starting from exon 2. Whereas 1TM receptors are already known for *OPRM1*, the new isoforms MOR-1TM1 and MOR-1TM2 will have unique C-termini and therefore add to the diversity of the receptor. Furthermore, MOR-1TM1, MOR-1TM2, and SV3 may also code for a 6TM receptor initiated from the first methionine on exon 2. The SV3 variant is of particular interest since it contains a known internal ribosome entry site (IRES) in exon 13 (Shabalina et al. 2009).

According to our prediction, six of the newly identified transcripts could code for 6TM receptors only: MOR-6TM1, MOR-1K3, MOR-1K4, MOR-1K5, MOR-1K6, and SV4. Five of these transcripts were obtained using a reverse primer in exon 13, which has been previously reported to be an alternative transcription start site and to connect only with exon 2 downstream. Interestingly, in five out of six cases the reverse priming from exon 13 yielded transcripts that include the exon 13 connected to other upstream exons (9b/c, 12, 14 and SVa/b/c/d), instead of being the first exon.

### Alternative Splicing of OPRD1 Resembles That of OPRM1

**Fig. 2:**
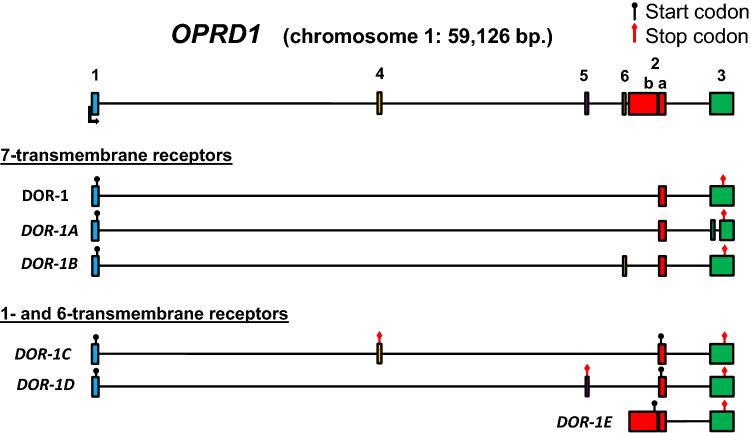
Human *OPRD1* gene structure and transcripts. Known exons and transcripts were identified using the UCSC browser and Ensembl. Known transcription start sites are marked with an angled arrow; known or putative start codons are marked with a black circle, and stop codons are marked with a red diamond. Modified from (Piltonen et al. 2019)

We next compared the alternative splicing pattern of *OPRM1 *with its closest evolutionary family member *OPRD1* (Stevens 2015). Multiple previously unknown splice variants of *OPRD1* have been described by our group in another publication (Piltonen et al. 2019, summarized in Fig. 2). The majority of these transcripts contain a cassette exon between exons 1 and 2, inducing either a PTC in DOR-1C and DOR-1D or adding 21 AAs to the first intracellular loop (ICL) in DOR-1B. Two transcripts contain a 5′ extension of exon 2 without exon 1 upstream, similar to *OPRM1* (µ3 and µ3-like). In general, the splicing patterns of *OPRD1* resemble those of *OPRM1* despite a substantial difference in the number of alternative transcripts, because both have transcripts that (a) have cassette exons between exons 1 and 2 inducing PTCs and subjecting the transcripts either to NMD or translation to 1TM and/or 6TM receptors, and (b) essentially start coding from exon 2 due to lack of coding exons upstream, yielding 6TM receptors.

### Alternative Splicing of *OPRK1* and *OPRL1* Reveal Common Patterns

**Fig. 3:**
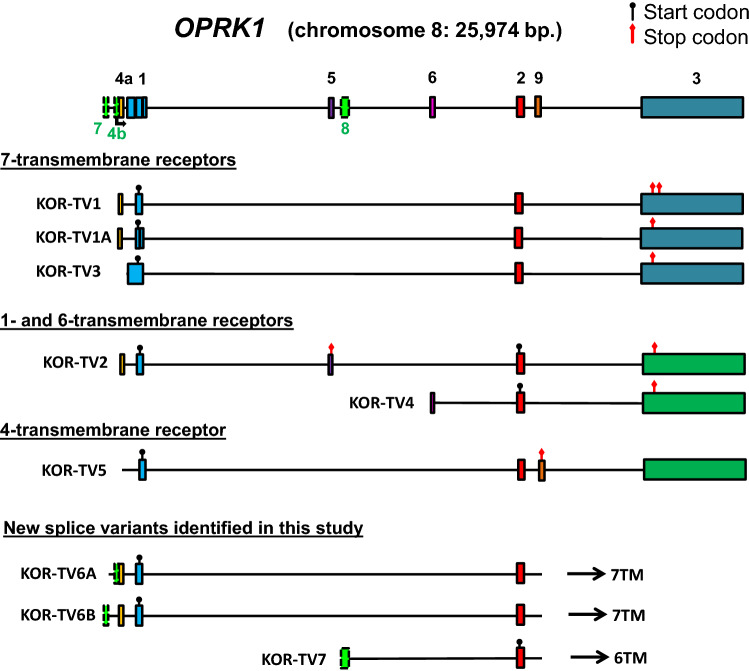
Human *OPRK1* gene structure and transcripts. Known exons and transcripts were identified using the UCSC browser and Ensembl. New exons discovered in this study are marked as green dashed-line boxes and named below the gene. Previously known exons are marked with solidly lined boxes filled with various colors for easy identification in the transcripts. Known transcription start sites are marked with an angled arrow; known or putative start codons are marked with a black circle, and stop codons are marked with a red diamond

We then analyzed two other members of OPR family members—*OPRK1* and *OPRL1.* Previously annotated *OPRK1* transcripts already represent the common set of full-length and truncated receptor isoforms (Fig. 3): three that code for 7TM receptors including one lacking 14 amino acids coded by exon 1 with a deletion (KOR-TV1A) and one with reported read-through of the termination codon to create variable C-terminal tails (KOR-TV1); one transcript for 1TM (KOR-TV2); and one transcript for 6TM-KOR (KOR-TV4). From the known transcripts, it appears that *OPRK1* has two transcription start sites: upstream of exon 1 and a possible a second site in the first intron since the TV4 transcript does not include exon 1. All the known transcripts apart from KOR-TV4 were represented in our human brain sample (Table 1), where we also identified an additional new exon upstream of exon 1 (KOR-TV6B) and an extension of exon 4 (KOR-TV6A), in the context of the canonical translation of a 7TM KOP (Fig. 3). We also found a transcript with a cassette exon (exon 8, KOR-TV7), which did not contain exon 1. Therefore, the new transcript KOR-TV7 resembles TV4 and is a putative source of a 6TM receptor isoform. It is noteworthy that we did not identify *OPRK1* 6TM variants with a 5′ extended exon 2, unlike in other OPRs. Also, rather surprisingly we did not detect *OPRK1* transcripts in BE(2)-C cells. Because the primers for 5′RACE were the same for the brain and BE(2)-C samples and the priming was efficient in the brain sample, this indicates that *OPRK1* was not expressed by BE(2)-C cells, and corroborates with previous findings (Standifer et al. 1994).

**Fig. 4:**
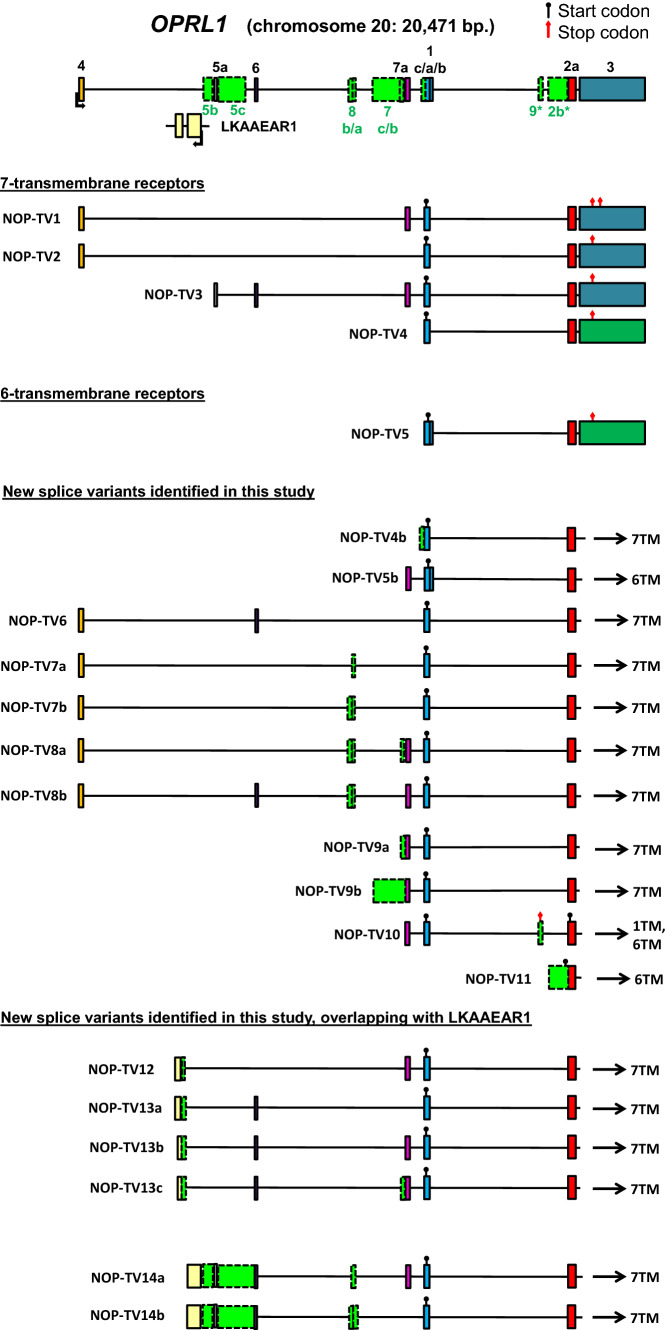
Human *OPRL1* gene structure and transcripts. Known exons and transcripts were identified using the UCSC browser and Ensembl. New exons discovered in this study are marked as green dashed-line boxes and named below the gene. Previously known exons are marked with solidly lined boxes filled with various colors for easy identification in the transcripts. Known transcription start sites are marked with an angled arrow; known or putative start codons are marked with a black circle, and stop codons are marked with a red diamond. Asterisks mark new cassette exons that are putatively coding

The known alternative splicing patterns of *OPRL1* contain many of the same elements as the other OPR genes. However, *OPRL1* seems to undergo extensive splicing upstream of exon 1, and our study confirms this (Fig. 4). All of the exons upstream of exon 1 appear to be non-coding, and do not change the N-terminus of the canonical 7TM NOP that they all encode. Also, similar to *OPRK1*, read-through of the conventional stop codon happens in exon 3 in NOP-TV1 (Loughran et al. 2014), and results in a unique C-terminal tail on the receptor. Thus far, read-through of the stop codon has not been reported for *OPRD1* or *OPRM1* suggesting that it may be a feature of *OPRK1* and *OPRL1* genes.

Interestingly, one of the 6TM variants of NOP does not result from skipping of exon 1 (NOP-TV5), but from the usage of an alternative translation start site inside exon 1 combined with an alternative splice donor site downstream of the conventional 3′ splice site of exon 1. The included part of exon 1 and its additional 3′ extension fail to fold into a TM domain and rather constitute a large intracellular N-terminus. We discovered a similar transcript, NOP-TV5b, but also another 6TM-coding variant with a 5′ extended exon 2 (NOP-TV11), which is similar to those seen in *OPRM1* and *OPRD1* as well. Therefore, *OPRK1* is the only family member where this type of transcript has not been reported but is very likely to exist; a transcript with extended exon 2 combined with exon 3 exists in mouse (GenBank AK138198). Our study also identified a 1TM variant coded by NOP-TV9 in the human brain, completing the typical selection of OPR isoforms.

Worth noting is that *OPRL1* shares exons with an overlapping gene on the opposite strand, *LKAAEAR1*. We found six transcripts, NOP-TV12, NOP-TV13a-c, and NOP-TV14a-b, which contain exons from the region of *LKAAEAR1*. Due to the sequencing technology capable of producing long reads up to several kb, we can reliably show that these elements are truly parts of single transcripts originating from the same strand. This opens a question of how *OPRL1* and *LKAAEAR1* transcripts with complementary sequences can potentially regulate each other.

### Evolutionary Conservation of OPR Alternative Transcription and Splicing Events for 6TM Variants

To verify the newly identified and previously established human exons in 6TM-coding transcripts through evolutionary conservation, we delineated the exact exon–intron boundaries and annotated them in different primate OPR genes using pairwise and multiple comparisons as well as evolutionary rate estimation (Table 2). Multiple comparisons were based on alignments of 100 vertebrate species that were retrieved for each exon separately along with a 100-nt extension on both ends from the UCSC Genome Browser (https://genome.ucsc.edu) and presented as PhastCons values in Table 2. We performed a detailed pairwise analysis of variable transcripts in human-macaque comparison to characterize the acquisition and evolution of novel alternative coding sequences by mammalian OPR genes. Comparative analysis of all four opioid receptor genes was performed based on the analysis of separate alternative and constitutive exons in the loci using slightly modified approach published earlier (Shabalina et al. 2010, 2014; Ogurtsov et al. 2008).

**Table 2:** Evolutionary comparison between human and macaque *OPRM1* (A), *OPRD1* (B), *OPRK1* (C) and *OPRL1* (D) constitutive exons, and exons found in 6TM-coding transcripts

	PhastCons Score*	lenQ	lens	lenA	K_u_/K_e_
*A) OPRM1*					
Exon11	0.217 ± 0.00113	523	535	523	0.044
*Exon1*	*0.272* ± *0.0009*	*732*	*732*	*732*	*0.035*
*Exon1/CDS*	*0.509* ± *0.0025*	*290*	*290*	*290*	*0.014*
*Exon2/CDS*	*0.931* ± *0.00135*	*353*	*353*	*351*	*0.014*
*Exon3/CDS*	*0.907* ± *0.00099*	*521*	*521*	*519*	*0.023*
*Exon4*	*0.073* ± *0.000003*	*13,672*	*13,479*	*12,811*	*0.058*
*Exon4/CDS*	*0.922* ± *0.01256*	*39*	*39*	*39*	*0.026*
Exon 2b	0.0774 ± 0.0078	64	66	64	0.083
Exon13	0.0724 ± 0.0004	1297	1290	1286	0.074
Exon SVa	0.02723 ± 0.0006	44	43	43	0
Exon SVb	0.0018 ± 0.0006	170	167	167	n/a
Exon6	0.0210 ± 0.0041	60	60	60	0
Exon7	0.0277 ± 0.0026	122	119	119	0.090
Exon8	0.0105 ± 0.0001	984	1050	983	0.067
Exon9b	0.0235 ± 0.0006	437	432	432	0.061
Exon9c	0.0005 ± 0.0003	123	123	123	0.078
Exon10	0.0168 ± 0.0024	73	73	72	0.073
Exon12	0.0170 ± 0.0003	766	761	758	0.080
Exon14a	0.0258 ± 0.0003	937	966	934	0.068
Exon14b	0.0215 ± 0.0025	77	80	77	0.040
ExonSVc	0.0415 ± 0.0006	662	682	661	0.067
ExonSVd^**^	0.01578 ± 0.0318	531	502	502	0.094
Exon13b	0.0169 ± 0.0003	969	990	967	0.057
B) *OPRD1*					
*Exon 1*	*0.308* ± *0.0014*	*469*	*468*	*468*	*0.042*
*Exon 1/CDS*	*0.579* ± *0.0029*	*227*	*227*	*227*	*0.036*
Exon 4	0.005 ± 0.0007	118	85	85	0.370
Exon 5	0.113 ± 0.0028	99	99	99	0.064
Exon 6/CDS	0.847 ± 0.0087	63	63	63	0.050
*Exon 2b*	*0.087* ± *0.0003*	*1656*	*911*	*911*	*0.079*
*Exon 2/CDS*	*0.905* ± *0.0015*	*350*	*350*	*350*	*0.015*
*Exon 3*	*0.136* ± *0.0001*	*8526*	*7648*	*7599*	*0.062*
*Exon 3/CDS*	*0.893* ± *0.0010*	*542*	*425*	*425*	*0.031*
C) *OPRK1*					
Exon4	0.022 ± 0.00137	249	238	238	0.085
*Exon1*	*0.577* ± *0.00022*	*305*	*305*	*305*	*0.041*
*Exon2/CDS*	*0.913* ± *0.00014*	*353*	*353*	*353*	*0.014*
*Exon3*	*0.157* ± *0.00001*	*4114*	*4115*	*4100*	*0.061*
*Exon3/CDS*	*0.805* ± *0.00011*	*534*	*534*	*534*	*0.028*
Exon5	0.008 ± 0.00090	174	107	107	0.058
Exon5/CDS	0.008 ± 0.00093	104	104	104	0.029
Exon6	0.003 ± 0.00066	215	215	215	0.048
Exon8	0.004 ± 0.00019	727	727	726	0.069
D) *OPRL1*					
Exon7a	0.050 ± 0.00285	151	178	150	0.048
*Exon1*	*0.449* ± *0.00232*	*284*	*284*	*284*	*0.047*
*Exon1/CDS*	*0.596* ± *0.00471*	*144*	*144*	*144*	*0.043*
*Exon2/CDS*	*0.812* ± *0.00017*	*356*	*356*	*356*	*0.026*
*Exon3*	*0.218* ± *0.00027*	*2468*	*2468*	*2499*	*0.072*
*Exon3/CDS*	*0.814* ± *0.00115*	*524*	*524*	*519*	*0.052*
Exon9	0.005 ± 0.00011	127	125	125	0.033
Exon2b	0.010 ± 0.00037	761	643	641	0.092

Constitutive exons are highlighted in italicized letters. lenQ = length of sequence human; lenS = length of sequence macaca; lenA = length of sequence considered for alignment; TT = number of transitions; TV = number of transversions; K_u_ or K_e_ = evolutionary rate (Kimura 2-parameter model)

*PhastCons Score is estimated based on multiple alignments of 100 vertebrate species downloaded from UCSC browser

^**^The length and alignment reflect only the unique nucleotides and not the repeated elements in the region

In all four OPR gene loci, the level of similarity in constitutive exons 2 and 3 was the highest, particularly in the coding regions with PhastCons values ranging from 0.805 to 0.931 (Table 2). The evolutionary rates (K_u_ or K_e_, both calculated using Kimura's two parameter model, see Materials and Methods) in the constitutive exons were also generally lower than in the alternative exons (Table 2). Most of the PhastCons values for alternative exons were low, ranging from 0.0005 to 0.217, which demonstrates that most of the alternative transcription events are conserved only in some primates or may even be human-specific. The intermediate level of conservation of exon 1, which codes for the first transmembrane domain, suggests it is an alternative exon rather than a constitutive one in all OPR loci. The sequence similarity of exon 1 is significantly higher than of newly discovered alternative exons yet notably lower than in exons 2 and 3. Nevertheless, this exon is ancient and belongs to the most common isoforms in all OPRs, in both humans and mice.

The high level of similarity between some human exons and several closely related primates including chimp and/or macaque can be explained by the fact that the exons are repeat-like sequences with high enrichment in the human genome (for example, see exon 4 in *OPRD1* gene). However, only few closely related primates contain these repeat-like sequences in orthologous positions.

### Translation of Transcripts Coding for 6TM OPR Variants

We used the GWIPS-Viz database (Michel et al. 2014) to find evidence that the newly discovered and known transcripts that code for 6TM and 1TM receptor variants were translated to create proteins. We investigated the alternative exons that are included in those transcripts and contain at least parts of the coding sequence. Most of the newly discovered exons in this study as well as some of the previously known exons are non-coding: since these sequences are not expected to be translated, they were excluded from the analysis. Also, the 6TM variants are usually translated starting from exon 2 and not from the upstream exons, making it impossible to distinguish the 6TM variants from full-length 7TM variants at the level of protein sequence. Finally, expression levels of the different receptor isoforms show large differences in the GWIPS-Viz dataset. *OPRM1* is particularly challenging due to lower expression levels in the datasets in GWIPS-Viz compared to the other OPRs. The only *OPRM1* 6TM transcripts for which we found evidence of translation were those containing exon 7 (Supplementary Fig. 3A), and even that only showed a few sporadic reads. The expression levels of *OPRD1* are slightly higher in the available datasets, and evidence for translation for 6TM and 1TM for DOPs have been reported previously (Piltonen et al. 2019).

In contrast, *OPRK1* and *OPRL1* both show strong gene expression levels and ribosomal footprints allowing reliable analysis. Thus, exon 5 of *OPRK1* had a robust ribosomal footprint at the beginning of the exon (Supplementary Fig. 2B), confirming the expression of the 1TM variant. This exon does not contain an AUG codon further downstream in the same frame with *OPRK1* coding sequence, and therefore the translation of the 6TM can restart only in exon 2. Because exon 2 is also found in the major isoform, it is not possible to separate the 6TM signal from the canonical 7TM signal. We also found evidence of translation for both 6TM receptor variants of *OPRL1* (Supplementary Figs. 2C, 3). The 3′ extension of exon 1, known as 1b, is found in NOP-TV5 and TV5b and shows a low level of ribosomal footprinting that also does not cover the whole length of the exon 1b. Exon 2b, which is an elongation of exon 2 at the 5′ end, has some ribosomal footprinting despite that there is no AUG start codon in that region. However, there is a potential alternative start codon CUG at the start of the footprinting similar to *OPRD1* variant DOR-1E (Piltonen et al. 2019).

### OPRs are Overrepresented Among the Rare N-Terminally Truncated 6TM GPCR Variants

Since all human OPR genes have transcript isoforms that can code for a 6TM receptor, we tested how unique the 6TM GPCR variants lacking the first TM domain are in the GPCR family. We searched the literature and screened the Uniprot database for human and mouse GPCR transcripts that would putatively code for either N- or C-terminally truncated 6TM variants (Table 3, Supplementary Table 1; 5TM added for comparison). According to our analysis, N-terminal 6TM truncations appear to be less frequent than C-terminal truncations: in this small group of only 12N-terminally truncated 6TM receptors, four of those are OPRs (Table 3).

**Table 3:** Human and mouse GPCR genes with annotated transcripts that can code for N-terminally truncated 6TM receptor variants that are implicated in pain, psychiatric disorders, and addiction

Receptor	Gene	Mouse	Human	Functionality of truncated receptor if known
Cholecystokinin CCK1 receptor	*CCKAR*	Q3TPL0		
Cholecystokinin CCK2 receptor	*CCKBR*		P32239-3	Lower affinity for ligands
Glucagon-like peptide 2 GLP-2 receptor	*GLP2R*	Q8BM22		
δ opioid receptor	*OPRD1*	MG986893*	MG986889*	
κ opioid receptor	*OPRK1*	AK138198.1*	P41145-2	
NOP receptor	*OPRL1*	B0R0C0	NM_001318855.1**	
μ opioid receptor	*OPRM1*	P42866-14	P35372-12	Exhibits excitatory cellular signaling; mediates analgesia, produces opioid-induced hyperalgesia, partially mediates tolerance
Olfactory receptor 2AJ1	*OR2AJ1*		A0A126GW62	
Olfactory receptor 56A5	*OR56A5*		A0A087WUB2	
Olfactory receptor 9Q2	*OR9Q2*		A0A126GVT3	
Pyroglutamylated RFamide Peptide Receptor	*QRFPR*		A1A4W1	
Tachykinin receptor 2	*TS′CR2*		A0A087WZ80	

Variant annotation codes from UniProt, except *GenBank accession, **RefSeq accession

### 6TM GPCRs are Enriched Among Genes Implicated in Pain, Psychiatric Disorders, and Addiction

**Fig. 5:**
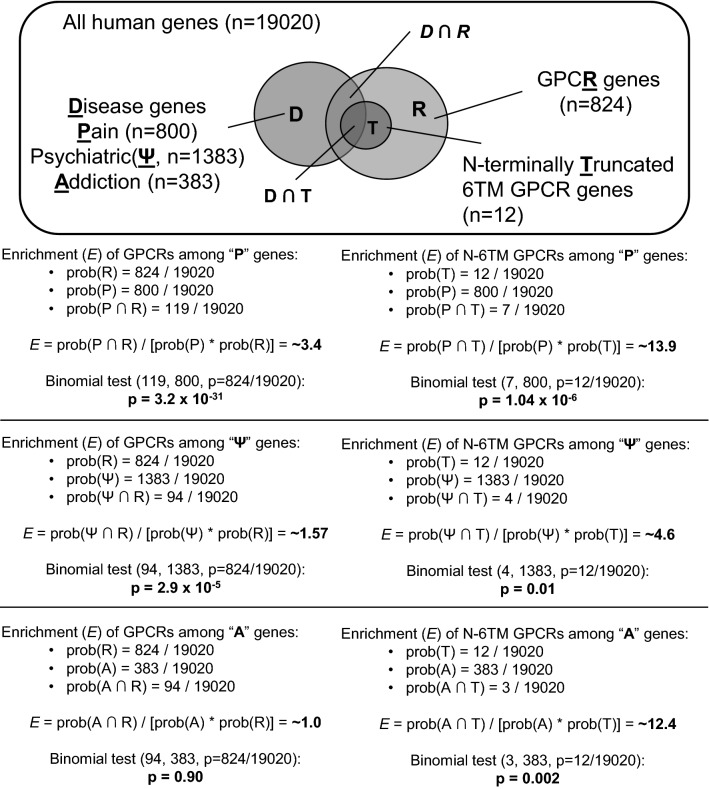
Enrichment of GPCR genes and N-terminally truncated 6TM GPCR genes among pain genes. There is a significant enrichment (*E*) of genes coding for 6TM GPCRs among genes related to pain, psychiatric disorders, and addiction when compared to their frequency in the human genome. Genes coding for GPCRs in general were enriched in pain genes and psychiatric disorder genes. Abbreviations: **A** = addiction-related genes, **P** = pain-related genes, **Ψ** = psychiatric disorder-related genes, **R** = GPCR genes, **T** = truncated 6TM GPCR genes

We then analyzed the possible enrichment for 6TM GPCRs isoforms among genes that are implicated in pain, psychiatric disorders, and addiction (Fig. 5, Supplementary Table 2). In this study, we found 12 GPCR genes that code for 6TM truncated variants (Table 3): 7 of these were found among the pain genes (*N* = 800), 4 among the genes related to psychiatric disorders (*N* = 1383), and 3 among the addiction-related genes (*N* = 383). Compared to their expected abundances in the human genome, 6TM GPCRs were significantly enriched in these three groups of disorder-related genes: 13.9-fold enrichment in the pain genes (binomial test *p* = 1.04 × 10^–6^), 4.6-fold enrichment in the psychiatric disorder genes (binomial test *p* = 0.01), and 12.4-fold enrichment in the addiction genes (binomial test *p* = 0.002). GPCR family genes in general were significantly enriched in the pain genes and psychiatric disorder genes, by approximately 3.4-fold and 1.6-fold, respectively (binomial test *p* = 3.2 × 10^–31^ and 2.9 × 10^–5^), but not in the addiction genes.

## Discussion

We have described an experimental survey of 5′ alternative splicing of transcript variants in the human OPR genes, namely *OPRM1*, *OPRD1, OPRK1*, and *OPRL1*. By creating a comprehensive map of OPR alternative splicing, we discovered 34 new transcripts expressed in the human brain and a human neuroblastoma cell line. In this study, we identified 10 previously unreported transcripts in *OPRM1,* three in *OPRK1,* and 17 in *OPRL1*, while four novel transcripts in *OPRD1* were reported in an earlier publication (Piltonen et al. 2019) and recapitulated here for convenience*.* The splicing pattern is analogous among OPR genes, and some common patterns can be observed in their splicing events.

Firstly, exons and alternative splice acceptor sites upstream of the first canonical coding exon (exon 1) either add amino acids to the N-terminal tail, or are non-coding, or lead to skipping of the first exon. These events are most prevalent in *OPRK1* and *OPRL1*.

Secondly, putative novel promoters and transcriptional start sites upstream of exon 2 produce transcripts for N-terminally truncated 6TM variants because of skipping of exon 1 and lack of replacing sequence that could substitute the missing first TM domain. This universal pattern of alternative transcription from potential promoters between exons 1 and 2 was found for all four human OPR genes, suggesting an ancient origin of alternative transcripts that code for 6TM isoforms of OPRs based on the variability of alternative promoters and transcription terminations (Shabalina et al. 2010, 2014). Specifically, comparison of human *OPRM1* and *OPRD1* shows conserved patterns of alternative transcription and splicing events between exons 1 and 2 despite the substantial difference between the frequencies of alternative transcripts: the number of alternative events in *OPRM1* is dramatically higher than in *OPRD1*. Comparative analysis of primate 6TM isoforms of *OPRM1*, *OPRD1*, and *OPRL1* supports the possibility that the alternative transcript starting from extended exons 2 (2b) could be translated from the conserved AUG/GUG/CUG in the frame with the canonical reading frame and code for 6TM protein isoforms with unique N-terminal tails. Most of the exon 2b sequence comprises non-coding 5′ UTR, where the overall conservation is much lower than in the 3′ end that produces the N-terminus (Shabalina et al. 2009; Piltonen et al. 2019). Some highly conserved elements can be found in the 5′UTR, consistent with the location of regulatory sites. For example, the newly discovered *OPRD1* exon 2b in mice is not highly conserved with humans or other primates. However, the very short 3′ tail of these exons is conserved and overlaps with potential 6TM open reading frames in primates (Piltonen et al. 2019). Interestingly, we did not observe an extended exon 2 in human *OPRK1* although it is present in the other OPR genes. Since such a transcript has been reported in the mouse *Oprk1,* it is likely to exist also in human but could be expressed in specific tissues and not captured in our experiment.

Finally, any alternative splicing events downstream of, and together with exon 1 usually introduce PTCs in all reading frames. These transcripts are putative sources of receptor fragments comprising only the first TM domain and N-terminal tail and may be subject to NMD. This also suggests that the newly discovered alternative events are involved in gene expression regulation through NMD (Piltonen et al. 2019; Maquat 2004), which can directly control transcript levels among other functions, especially in the brain (D'Lima et al. 2017; Yan et al. 2015; Aebersold et al. 2018). *OPRM1* shows the largest number of this type of cassette exon inclusions, but they are typical for *OPRD1* as well. As an exception, we previously reported a rare event in *OPRD1* where the cassette exon does not interrupt the reading frame from exon 1 but inserts 21 AAs in the first intracellular loop of a full 7TM receptor (Fig. 2, transcript DOR-1B) (Piltonen et al. 2019). The majority of transcripts in this category (those that contain exons 2 and 3 downstream, see Figs. 1, 2, 3, and 4) can simultaneously code for 6TM isoforms from the first methionine at the beginning of exon 2, potentially supporting the idea that the evolution of NMD exons and 6TM isoforms are concomitant events. Especially interesting in this regard is *OPRM1* transcript SV3 which has exon 1 and a PTC in exon SVa to produce a 1TM fragment, and downstream it has the components of MOR-1K isoforms: importantly, exon 13 containing an IRES and can therefore initiate the translation of a 6TM receptor (Shabalina et al. 2009). It is also worth noting that even though the splice variants coding for 6TM receptors have non-coding exons at the 5′ end, this classification only means that they do not code for a transmembrane helix or an additional peptide tail integrated to the receptor. They may still code for short peptides from the uORFs, upstream open reading frames. The expression and functions of such small peptides is still poorly understood, but they may be important regulators of the translation process, protein–protein interactions, and cell communications (Cabrera-Quio et al. 2016).

As we have discussed above, the splicing events are similar between the OPR genes, but alternative exons however are generally not well conserved between species. The coding regions defined by exons 2 and 3, essentially forming the N-terminally truncated 6TM receptor variants, are the most conserved regions in each OPR gene. Interestingly, exon 1 in all OPR genes is much less conserved also in the coding region and could even be considered an alternative exon despite that it is the source of the first transmembrane domain in the canonical, full-length OPRs. Thus, the evolutionary pressure seems to support 6TM variants through a variety of mechanisms, but not necessarily through any specific exon.

The similarity in the gene structure and transcription events within the OPR family likely arises from the evolutionary path of the four genes. Apparently they evolved from a single ancestral opioid receptor gene through two rounds of genome duplication, of which the first one produced the MOP/DOP and KOP/NOP ancestors (Stevens et al. 2007; Dreborg et al. 2008). These ancestral pairs are both supported and contrasted in the splicing patterns as described in the previous paragraph: MOP has been assigned as the fast-evolving receptor and DOP as the slow evolving receptor of the MOP/DOP ancestor, which is reflected in the number of alternatively spliced transcripts (Stevens et al. 2007). As we confirmed in this study, the number of alternative events is significantly higher for *OPRM1* than for any other OPR. For example, the number of 6TM-coding transcripts is 3–4 times higher in *OPRM1* than in other OPRs. Specifically, we identified 16 potential 6TM transcript variants in *OPRM1*, three in *OPRD1* and *OPRK1*, and four in *OPRL1*. Since some of these splice variants show different ligand binding or signaling pathways and the highest level of SNP density in human *OPRM1* gene locus (Stevens 2009; Pan 2014), the enrichment of alternative events in human *OPRM1* gene locus may support the hypothesis that *OPRM1* differs from the other OPRs by higher measurable evolutionary pressure and presence of signature of adaptive evolution in the gene locus.

We have presented evidence for translation of some of the 6TM-producing transcripts by examining ribosomal footprinting of OPR genes. This analysis is only possible when a unique coding region exists in the transcript, as any signal that overlaps with exons 1, 2, and 3 will be overwhelmed by the expression of the major transcripts and other receptor variants. The clearest evidence for translation of 6TM receptors was found in *OPRL1*, where we observed ribosomal signals in exons 1b and 2b. Translation in the absence of a canonical AUG start codon in exon 2b implies that an alternative start codon CUG at the start of the footprinting is used. We observed a similar phenomenon in the mouse *Oprd1* variant DOR-1E (Piltonen et al. 2019). As discussed above, this appears to be a conserved origin for 6TM receptors in the OPR family.

Observing such a rich presentation of 6TM receptor variants among OPRs, we expected it to be a common event among the GPCRs. Altogether, we expanded our search to include transcripts missing the first or last 1–2 helices among GPCRs to cover N- and C-terminally truncated 6TM receptors, as well as 5TM receptors for comparison (Supplementary Table 1). Our analysis showed that the 6TM N-terminally truncated variants were relatively rare: only 12 of those variants were identified in human, four of which were OPRs (Table 3). Since OPRs play roles in pain perception, some psychiatric disorders and addiction (Lutz and Kieffer 2013; Calo et al. 2000; Mercadante and Romualdi 2020; Pradhan et al. 2011), we examined the presentation of these group of 6TM receptors in the same categories. We discovered a significant enrichment of 6TM-coding genes in all three categories of disorder-related genes. Since this analysis was focused on a limited number of psychiatric and neurological disorders, we cannot exclude the possibility that 6TM receptors may be relevant in many other diseases and health conditions. However, our analysis shows that broadening the spectrum of pharmacological research and drug discovery to include the less known receptor variants may hold some promise for novel discoveries in these disorders—pain, psychiatric disorders, and addiction—that are typically challenging to treat. Of note, our study shows that the expression of 1TM and 6TM OPR variants is dependent on alternative transcription initiations or/and terminations and cannot be described by alternative splicing alone. Thus, alternative promoters or enhancers are important in defining the transcript structures, and genetic variation in these promoter/enhancer regions may alter the expression of OPR variants. This may help to explain individual differences in phenomena like pain sensitivity (Shabalina et al. 2009) and highlights the importance of studying the molecular mechanisms of truncated receptor variant expression.

We have focused on 6TM GPCRs in the scope of this study because they represent the only truncated GPCR variants with largely intact ligand-binding pockets. The N-terminus is an important functional domain, which contains post-translational modification sites, participates in receptor trafficking and expression on the plasma membrane, and is an integral part of receptor activation in certain GPCR families through multiple mechanisms (see Coleman et al. (2017) for a review on N-terminus). However, in rhodopsin-type GPCRs (such as the OPRs) the N-terminus and the first TM domain are not known to contact ligands directly, and may be omitted from the receptor structure without a significant effect on the ligand binding. Therefore, N-terminally truncated 6TM receptor variants can be functionally relevant as they are likely to bind the same ligands as the full-length receptors but retain signaling capacity since they still contain the intracellular domains and the C-terminal tail. However, the signaling can drastically differ from the full-length isoform. For example, the unique functionality of the 6TM MOP has been reported in several studies. Activation of the 6TM MOP leads to release of nitric oxide and accumulation of intracellular calcium in transiently transfected BE(2)-C neuroblastoma cells, which are excitatory rather than inhibitory events and in contrast to the canonical 7TM MOP signaling (Gris et al. 2010; Samoshkin et al. 2015; Convertino et al. 2015b). Finally, animal experiments have shown that 6TM MOPs play distinct role in opioid analgesia, opioid tolerance, dependence, and opioid-induced hyperalgesia (Samoshkin et al. 2015; Marrone et al. 2017; Majumdar et al. 2011; Lu et al. 2015), and sometimes these roles can be also opposite to those of 7TM MOP (Samoshkin et al. 2015).

The skepticism regarding the functionality of N-terminally truncated 6TM GPCRs is related to the fact that the N-terminus, absent in such variants, typically participates in directing a receptor to the cell surface during receptor maturation, among other diverse functions. Thus, it is no surprise that such truncated GPCRs are typically located intracellularly. However, it is now well established that GPCRs can signal from intracellular compartments as well, including endosomal and Golgi membranes, with a significant level of contribution to the overall signaling response (Irannejad et al. 2013, 2017; Jong et al. 2018). In the case of OPRs, opioid peptides and small-molecule opioids have been shown to affect the intracellular and cell surface receptor pools in a very different manner (Stoeber et al. 2018). Whereas opioid peptides signal from the plasma membrane receptors, and the internalized receptors from endosomes, drugs like morphine and etorphine additionally cause a rapid activation of receptors in Golgi. In line with the general intracellular location of truncated receptors, it was shown that if 6TM MOP is transiently expressed in cells alone, it is mainly located inside the cell and poorly localized to the plasma membrane (Gris et al. 2010; Samoshkin et al. 2015; Majumdar et al. 2011). However, the expression on the cell surface increases dramatically when the beta2-adrenoceptor or NOP is co-expressed with 6TM MOP, and also leads to enhanced functional effects (Samoshkin et al. 2015; Majumdar et al. 2011). Therefore, the effects and roles of truncated receptors can be complex and dependent on the tissue or cell type where they are expressed, and the availability of a chaperone receptor.

Finally, we acknowledge that much shorter receptor variants or fragments that can exhibit specific functional effects have been discovered, including the 1TM MOP which stabilizes the full-length MOP and thus participates in opioid analgesia (Xu et al. 2013). Our 5′RACE experiment was designed to focus on the discovery of 6TM isoforms of the OPRs and does not exclude the possibility that additional receptor variants exist. Indeed, a transcript coding for a C-terminally truncated 4TM KOP has been reported to arise from a combination of alternative splicing events: the use of alternative splice donor and acceptor sites at exons 2 and 3, and an insertion of cassette exon between exons 2 and 3 (Gaveriaux-Ruff et al. 1997). The function of this receptor variant remains unknown.

In conclusion, we have explored the alternative splicing of the 5′ ends of OPR transcripts and discovered several previously unknown transcripts potentially coding for 1TM, 6TM, or 7TM receptor variants. We identified a conserved pattern of alternative splicing for this receptor family, suggesting evolutionary pressures supporting the evolving of these receptors, including these truncated variants. Whereas the splicing patterns are very similar between the genes, the alternatively spliced exons show a relatively low conservation between vertebrate species. The primary structures of alternative transcripts may be deterministic in each aspect of potential opioid function including ligand binding, conformational change, signal transduction, and post-translational regulation. Our analysis also shows that the N-terminally truncated 6TM receptors, common in the OPR family, are rare in the GPCR superfamily. These 6TM receptor variants are also overrepresented in genes related to pain, some psychiatric disorders, and addiction. The expanding evidence on the functionality and the specific roles played by 6TM OPRs calls for more research on other 6TM GPCRs as well, and may even help to find novel solutions for the development of new medical therapies.

## Electronic supplementary material

Below is the link to the electronic supplementary material.

Supplementary file1 (XLSX 44 kb)

Supplementary file2 (DOCX 2286 kb)
