# Correction to: Alternative Splicing of Opioid Receptor Genes Shows a Conserved Pattern for 6TM Receptor Variants

**DOI:** 10.1007/s10571-020-00999-9

**Published:** 2020-11-17

**Authors:** Marjo Piltonen, Andrey Krokhotin, Marc Parisien, Pierre Bérubé, Haig Djambazian, Rob Sladek, Nikolay V. Dokholyan, Svetlana A. Shabalina, Luda Diatchenko

**Affiliations:** 1 School of Dentistry, McGill University, Genome Building, Room 2201, 740 Dr. Penfield Avenue, Montreal, Quebec H3A 0G1 Canada; 2 Department of Anesthesia, School of Medicine, McGill University, Genome Building, Room 2201, 740 Dr. Penfield Avenue, Montreal, Quebec H3A 0G1 Canada; 3 Alan Edwards Centre for Research on Pain, McGill University, Genome Building, Room 2201, 740 Dr. Penfield Avenue, Montreal, Quebec H3A 0G1 Canada; 4 Departments of Pathology, Genetics and Developmental Biology, Stanford Medical School, Howard Hughes Medical Institute, Palo Alto, CA 94305 USA; 5 Departments of Human Genetics and Medicine, Faculty of Medicine, McGill University, Montreal, Quebec H3A 0G1 Canada; 6 McGill University and Génome Québec Innovation Centre, Montreal, Quebec H3A 0G1 Canada; 7 Departments of Pharmacology, and Biochemistry & Molecular Biology, Penn State College of Medicine, Hershey, PA 17033-0850 USA; 8 Departments of Chemistry, and Biomedical Engineering, Penn State, University Park, PA 16802 USA; 9 National Center for Biotechnology Information, National Library of Medicine, National Institutes of Health, Building 38A, Room S604, 8600 Rockville Pike MSC 3830, Bethesda, MD 20894-6075 USA

## Correction to: Cellular and Molecular Neurobiology 10.1007/s10571-020-00971-7

The article “Alternative Splicing of Opioid Receptor Genes Shows a Conserved Pattern for 6TM Receptor Variants”, written by Marjo Piltonen, Andrey Krokhotin, Marc Parisien, Pierre Bérubé, Haig Djambazian, Rob Sladek, Nikolay V. Dokholyan, Svetlana A. Shabalina and Luda Diatchenko was originally published electronically on the publisher’s internet portal on October 3, 2020 without open access. With the author(s)’ decision to opt for Open Choice the copyright of the article changed on October 27, 2020 to © The Author(s) 2020 and the article is forthwith distributed under a Creative Commons Attribution 4.0 International License, which permits use, sharing, adaptation, distribution and reproduction in any medium or format, as long as you give appropriate credit to the original author(s) and the source, provide a link to the Creative Commons licence, and indicate if changes were made. The images or other third party material in this article are included in the article’s Creative Commons licence, unless indicated otherwise in a credit line to the material. If material is not included in the article’s Creative Commons licence and your intended use is not permitted by statutory regulation or exceeds the permitted use, you will need to obtain permission directly from the copyright holder. To view a copy of this licence, https://creativecommons.org/licenses/by/4.0/.

