# Supplementary material for: Alternative Splicing of Opioid Receptor Genes Shows a Conserved Pattern for 6TM Receptor Variants
Source: Cell Mol Neurobiol. 2020 Oct 3;41(5):1039–55. doi: 10.1007/s10571-020-00971-7 (PMC8159799; doi:10.1007/s10571-020-00971-7)
Supplement: Supplementary file 2 — Supplementary file2 (DOCX 2286 kb) [file 10571_2020_971_MOESM2_ESM.docx]

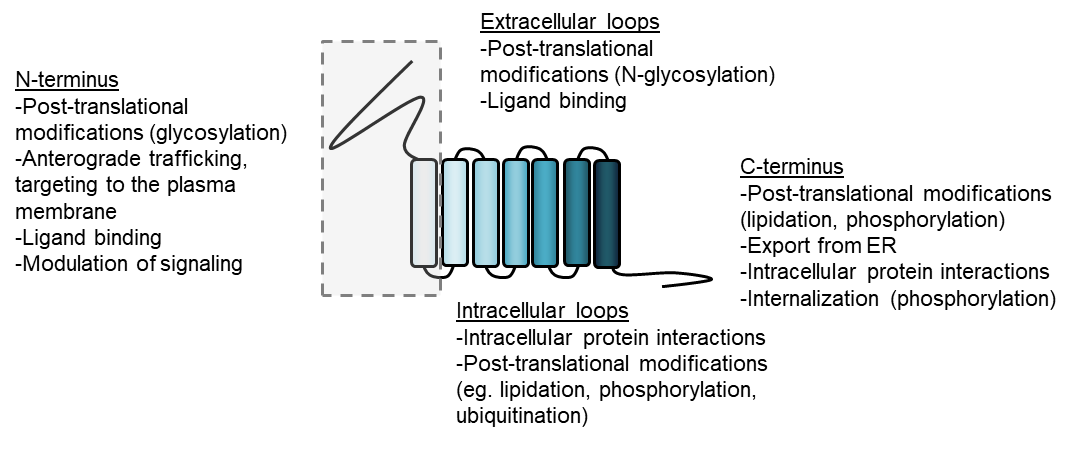


**Supplementary Figure 1.** General structure and functional domains of a full-length 7TM GPCR. In N-terminally truncated 6TM receptors, first transmembrane domain is missing (box) and sometimes replaced by an elongated intracellular tail instead of the first intracellular loop.


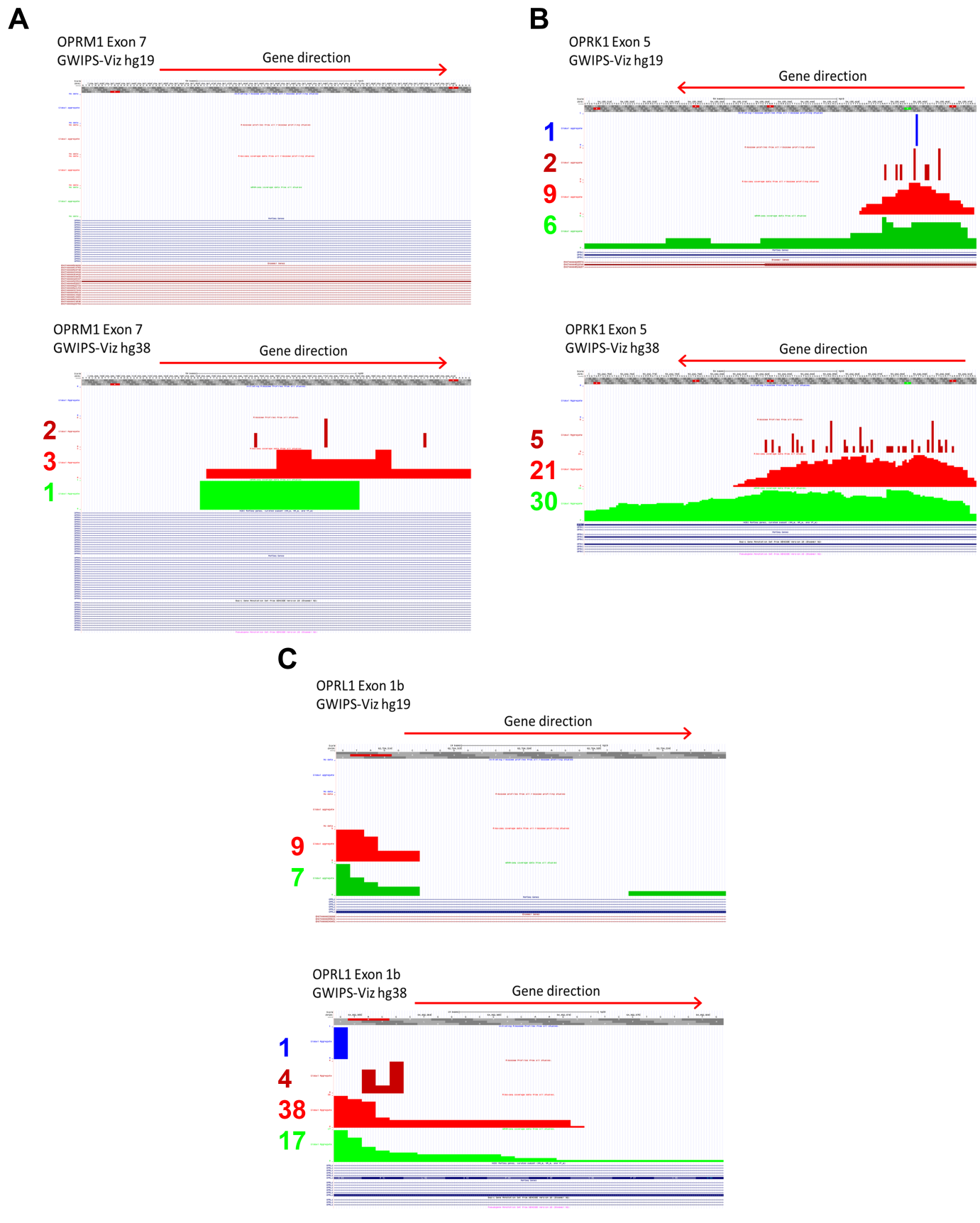


**Supplementary Figure 2.** Ribosomal footprints in 6TM variant-related exons of *OPRM1, OPRK1* and *OPRL1*. Data from GWIPS-Viz browser, depicting sequencing read pileups of ribosomal footprints in both hg19 and hg38. The height of the track (maximum number of reads) is expressed as numbers on the left with the color matching the color of the track: Blue = All Initiating Ribosomes (P-site) tracks), dark red = All Elongating Ribosomes (A-site) tracks), red = All Elongating Ribosomes (Footprints) tracks, green = All mRNA-seq Reads tracks. **A)** Ribosomal footprints on *OPRM1* cassette exon 7 show a few reads falling in this area, corresponding to the low expression of MOR-1TM1 in our experiments. **B)** Ribosomal footprints on *OPRK1* cassette exon 5 found in KOR-TV2. The ribosomal footprints at the start of the exon (not the reversed gene direction) confirm the expression of the 1TM fragment, but the 6TM variant would putatively start from exon 2. **C)** Some ribosomal footprints on *OPRL1* exon 1b (3’ extension of canonical exon 1) found in NOP-TV5 and TV5b. Figures enlarged and annotated from screenshot from the GWIPS-Viz browser.

**
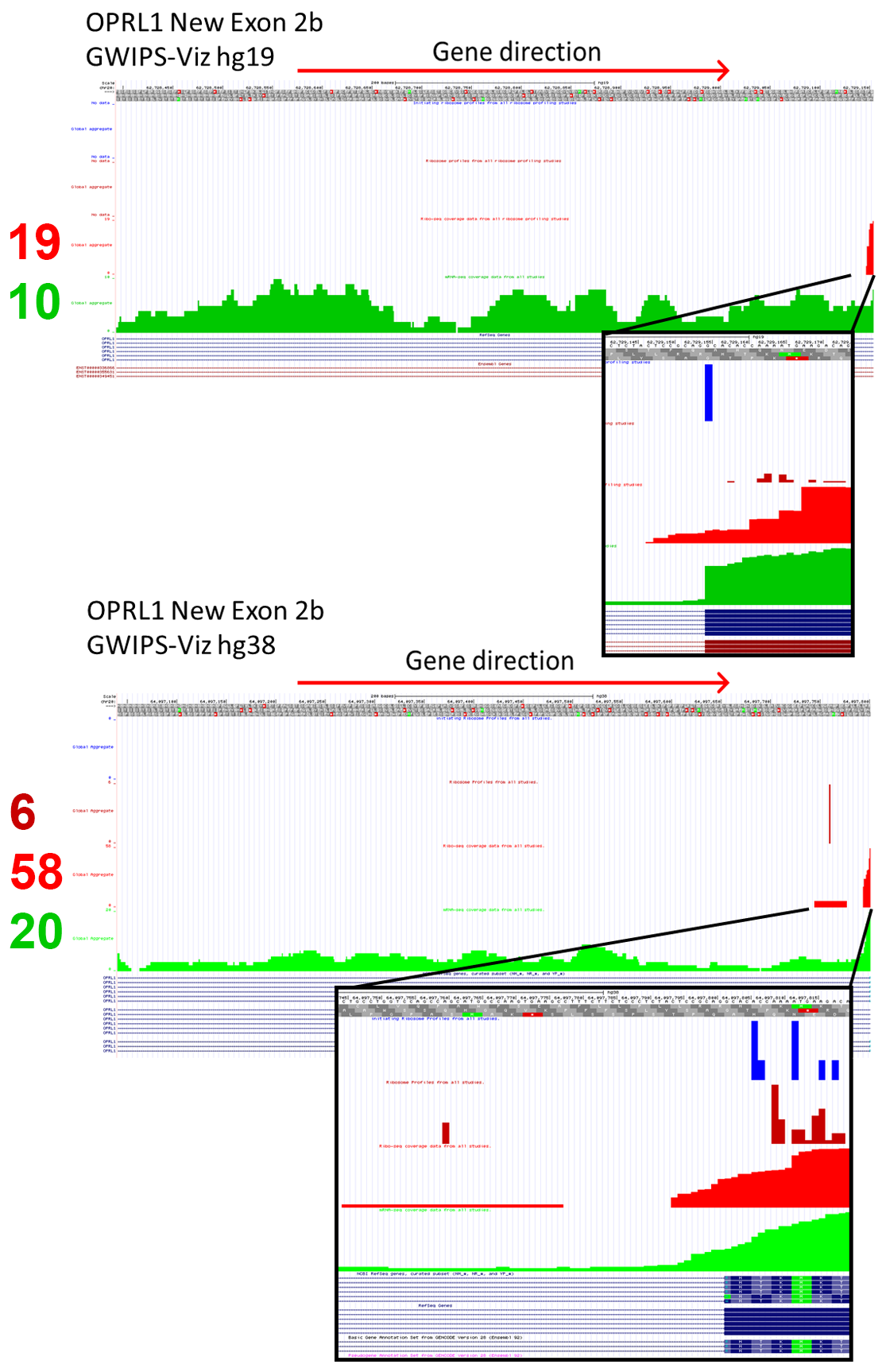
**

**Supplementary Figure 3.** Ribosomal footprints in exon 2b (NOP-TV11, 6TM variant) in *OPRL1*. Ribosomal footprints extend to exon 2b indicating a translation start site within this exon. Due to the lack of a canonical AUG start codon in this area, the translation is likely to start from an alternative codon such as CUG at the beginning of the footprint. The zoom-in boxes contain also the beginning of exon 2, where a second putative translation start site, a canonical AUG, is noted with a green arrow. Data from GWIPS-Viz browser, depicting sequencing read pileups of ribosomal footprints in both hg19 and hg38. The height of the track (maximum number of reads) is expressed as numbers on the left with the color matching the color of the track: Blue = All Initiating Ribosomes (P-site) tracks), dark red = All Elongating Ribosomes (A-site) tracks), red = All Elongating Ribosomes (Footprints) tracks, green = All mRNA-seq Reads tracks.

**Supplementary Table 1**. Human and mouse GPCR genes with annotated transcripts that can code for C-terminally truncated 6TM, and N- and C-terminally truncated 5TM receptor variants (from UniProt, with variant annotation code).

| **6TM truncated GPCRs** | | | | |
| --- | --- | --- | --- | --- |
| **7th HELIX MISSING = C-terminally truncated 6TM receptor** | | | | |
| **Receptor** | **Gene** | **Mouse** | **Human** | **Functionality of truncated receptor if known** |
| Adhesion G Protein-Coupled Receptor G4 | *ADGRG4* |  | Q8IZF6-2 |  |
| α1A-adrenoceptor | *ADRA1A* | Q8BXZ4 | P35348-5 | not functional alone; membrane expression and activity of WT receptor ↓ [1] |
| Vasopressin V2 receptor | *AVPR2* |  | P30518-2 |  |
| Calcitonin CT receptor | *CALCR* |  |  | Similar receptor in rabbit: membrane expression and activity of WT receptor ↓ [2] |
| Frizzled-6 | *FZD6* |  | G5EA13 |  |
| Glucagon receptor | *GCGR* |  | I3L454X |  |
| Growth-hormone-releasing hormone receptor (GHRHR) | *GHRHR* | A0A0N4SVS4 |  |  |
| G-protein-coupled receptor GPR6 (orphan) | *GPR6* |  | E7EP76 |  |
| G-protein-coupled receptor GPR78 (orphan) | *GPR78* |  | D6RB95 |  |
| G-protein-coupled receptor GPR158 (orphan) | *GPR158* | Q8BSU1 |  |  |
| Metabotropic glutamate mGlu4 receptor | *GRM4* | Q68EF4-2 |  |  |
| Metabotropic glutamate mGlu7 receptor | *GRM7* |  | B7ZKK0 |  |
| Hypocretin (Orexin) Receptor 2 | *HCRTR2* |  | S4WX84 |  |
| Histamine H3 receptor | *HRH3* | E9Q540 |  | Similar receptor in rat: not functional alone; membrane expression and activity of WT receptor ↓ [3] |
| Neuropeptide Y Receptor Y1 | *NPY1R* | Q04573-2 |  |  |
| Melanocortin 4 receptor | *MC4R* |  | A0N0W8 |  |
| Mas-related G-protein coupled receptor member A6 | *MRGPRA6* | A0A087WQV0 |  |  |
| Olfactory receptor 4X1 | *OR4X1* |  | A0A126GVZ7 |  |
| Olfactory receptor 5D16 | *OR5D16* |  | A0A126GW99 |  |
| Olfactory receptor 180 | *OLFR180* | A0A0U1RNK4 |  |  |
| Olfactory receptor 998 | *OLFR998* | B2RWV2 |  |  |
| Prostanoid DP1 receptor | *PTGDR* |  | Q13258-2 |  |
| Prostanoid FP receptor | *PTGFR* |  | P43088-2 | functionally inactive, perinuclear localization [4] |
| Prostanoid IP receptor | *PTGIR* |  | A0A0B4J2A7 |  |
| Retinal G protein coupled receptor | *RGR* | A0A286YCJ5 |  |  |
| Taste receptor type 2 member 46 | *TAS2R46* |  | A0A0J9YY42 |  |
| **5TM truncated GPCRs** | | | | |
| **1&2 HELICES MISSING** | | | | |
| **Receptor** | **Gene** | **Mouse** | **Human** | **Functionality of truncated receptor if known** |
| Adhesion G protein-coupled receptor E1 | *ADGRE1* |  | Q14246-2 |  |
| Cholecystokinin CCK2 receptor | *CCKBR* |  | only in literature | not functional alone; membrane expression and activity of WT receptor ↓ [5] |
| Chemokine receptor type 5, CCK5 | *CCR5* |  | only in literature | membrane expression of WT receptor ↓ (ccr5Δ32, [6]); normal expression & functionality for another 5TM variant [7] |
| Chemokine receptor type 8, CCK8 | *CCR8* |  | P51685-2 |  |
| Corticotropin-releasing factor CRF1 receptor | *CRHR1* |  | P34998-5 |  |
| F2R Like Thrombin/Trypsin Receptor 3 | *F2RL3* | Q0VDX1 |  |  |
| G-protein-coupled receptor GPR161 (orphan) | *GPR161* |  | Q8N6U8-5 |  |
| G-protein-coupled receptor GPR143 (orphan) | *HTR2A* |  | P28223-2 |  |
| Sphingosine-1-phosphate receptor 1 | *S1PR1* | Q8C4A3 |  |  |
| **6&7 HELICES MISSING** | | | | |
| **Receptor** | **Gene** | **Mouse** | **Human** | **Functionality of truncated receptor if known** |
| α2C-adrenoceptor | *ADRA2C* |  | D6RGL0 |  |
| Vasopressin V2 receptor | *AVPR2* | Q3SWS4 | only in literature | Non-functional receptors incapable of binding ligands and signal transduction. Functional rescue of mutant V2 vasopressin receptors causing nephrogenic diabetes insipidus by a co-expressed receptor polypeptide.  [8] |
| Bradykinin B1 receptor | *BDKRB1* |  | G3V4Y2 |  |
| Cholecystokinin CCK2 receptor | *CCKBR* | Q8BYG7 |  |  |
| Dopamine D3 receptor | *DRD3* |  | only in literature | not functional alone; membrane expression and activity of WT receptor ↓ [9,10] |
| Free fatty acids FFA4 receptor | *FFAR4* |  | S4R3L2 |  |
| Growth-hormone-releasing hormone receptor (GHRHR) | *GHRHR* |  | only in literature | not functional alone; signal transduction of WT receptor ↓ [11] |
| Ghrelin receptor | *GHSR* |  | Q92847-2 | not functional alone; membrane expression and activity of WT receptor ↓ or ↑ when expression of alternative receptor is high or low, respectively [12-14] |
| Gonadotrophin-releasing hormone GnRH1 receptor | *GNRHR* | Q6P8H4 | only in literature | not functional alone; membrane expression and activity of WT receptor ↓ [15] |
| G-protein-coupled receptor GPR39 (orphan) | *GPR39* | A6QR71 | A0A087WTL7 |  |
| G-protein-coupled receptor GPR107 (orphan) | *GPR107* | Q3UQH1 |  |  |
| Histamine H3 receptor | *HRH3* | E9Q522 | Q8NI49 |  |
| Luteinizing hormone LH receptor | *LHCGR* |  | only in literature | Binds ligands poorly, no signal transduction [16] |
| Motilin receptor | *MLNR* |  | O43193-2 |  |
| Neuromedin B receptor | *NMBR* | A0A087WP36 |  |  |
| Neuropeptide Y1 receptor | *NPY1R* |  | only in literature | [17] |
| Neurotensin NTS2 receptor | *NTSR2* | A0A1Y7VN85 |  | Similar receptor in rat: mostly intracellular localization, is functional and forms dimers with WT receptor [18] |
| Olfactory receptor 10D3 | *OR10D3* |  | A0A126GVM5 |  |
| Short-wave-sensitive opsin 1 | *OPN1SW* | G3UXM0 |  |  |
| Pyroglutamylated RFamide Peptide Receptor | *QRFPR* | A0A0G2JH05 | J3KNR3 |  |
| Retinal G protein coupled receptor | *RGR* |  | P47804-3 |  |
| Somatostatin SST5 receptor | *SSTR5* |  | D2CFK4 | mostly intracellular localization, is functional and shows ligand selectivity [19] |
| Smoothened | *SMO* | Q80VD0 |  |  |
| Sphingosine-1-phosphate receptor 5 | *S1PR5* |  | Q9H228-2 |  |
| Transmembrane Protein Adipocyte Associated 1 | *TPRA1* | A0A0N4SW75 | Q86W33-2 |  |

**References**

1. Coge F., Guenin S. P., Renouard-Try A., Rique H., Ouvry C., Fabry N., Beauverger P., Nicolas J. P., Galizzi J. P., Boutin J. A. and Canet E., (1999) Truncated isoforms inhibit [3H]prazosin binding and cellular trafficking of native human alpha1A-adrenoceptors. Biochem. J. 343 Pt 1, 231-239. DOI:10.1042/0264-6021:3430231

2. Seck T., Pellegrini M., Florea A. M., Grignoux V., Baron R., Mierke D. F. and Horne W. C., (2005) The delta e13 isoform of the calcitonin receptor forms a six-transmembrane domain receptor with dominant-negative effects on receptor surface expression and signaling. Mol. Endocrinol. 19, 2132-2144. DOI:me.2004-0472

3. Bakker R. A., Lozada A. F., van Marle A., Shenton F. C., Drutel G., Karlstedt K., Hoffmann M., Lintunen M., Yamamoto Y., van Rijn R. M., Chazot P. L., Panula P. and Leurs R., (2006) Discovery of naturally occurring splice variants of the rat histamine H3 receptor that act as dominant-negative isoforms. Mol. Pharmacol. 69, 1194-1206. DOI:mol.105.019299

4. Vielhauer G. A., Fujino H. and Regan J. W., (2004) Cloning and localization of hFP(S): a six-transmembrane mRNA splice variant of the human FP prostanoid receptor. Arch. Biochem. Biophys. 421, 175-185. DOI:S0003-9861(03)00591-5

5. Sanchez C., Escrieut C., Clerc P., Gigoux V., Waser B., Reubi J. C. and Fourmy D., (2012) Characterization of a novel five-transmembrane domain cholecystokinin-2 receptor splice variant identified in human tumors. Mol. Cell. Endocrinol. 349, 170-179. DOI:10.1016/j.mce.2011.10.010 [doi].

6. Benkirane M., Jin D. Y., Chun R. F., Koup R. A. and Jeang K. T., (1997) Mechanism of transdominant inhibition of CCR5-mediated HIV-1 infection by ccr5delta32. J. Biol. Chem. 272, 30603-30606. DOI: 10.1074/jbc.272.49.30603

7. Ling K., Wang P., Zhao J., Wu Y. L., Cheng Z. J., Wu G. X., Hu W., Ma L. and Pei G., (1999) Five-transmembrane domains appear sufficient for a G protein-coupled receptor: functional five-transmembrane domain chemokine receptors. Proc. Natl. Acad. Sci. U. S. A. 96, 7922-7927. DOI: 10.1073/pnas.96.14.7922

8. Schoneberg T., Yun J., Wenkert D. and Wess J., (1996) Functional rescue of mutant V2 vasopressin receptors causing nephrogenic diabetes insipidus by a co-expressed receptor polypeptide. EMBO J. 15, 1283-1291. DOI:10.1002/j.1460-2075.1996

9. Karpa K. D., Lin R., Kabbani N. and Levenson R., (2000) The dopamine D3 receptor interacts with itself and the truncated D3 splice variant d3nf: D3-D3nf interaction causes mislocalization of D3 receptors. Mol. Pharmacol. 58, 677-683. DOI:10.1124/mol.58.4.677

10. Elmhurst J. L., Xie Z., O'Dowd B. F. and George S. R., (2000) The splice variant D3nf reduces ligand binding to the D3 dopamine receptor: evidence for heterooligomerization. Brain Res. Mol. Brain Res. 80, 63-74. DOI:S0169328X00001200

11. Motomura T., Hashimoto K., Koga M., Arita N., Hayakawa T., Kishimoto T. and Kasayama S., (1998) Inhibition of signal transduction by a splice variant of the growth hormone-releasing hormone receptor expressed in human pituitary adenomas. Metabolism. 47, 804-808. DOI:S0026-0495(98)90116-0

12. Howard A. D., Feighner S. D., Cully D. F., Arena J. P., Liberator P. A., Rosenblum C. I., Hamelin M., Hreniuk D. L., Palyha O. C., Anderson J., Paress P. S., Diaz C., Chou M., Liu K. K., McKee K. K., Pong S. S., Chaung L. Y., Elbrecht A., Dashkevicz M., Heavens R., Rigby M., Sirinathsinghji D. J., Dean D. C., Melillo D. G., Patchett A. A., Nargund R., Griffin P. R., DeMartino J. A., Gupta S. K., Schaeffer J. M., Smith R. G. and Van der Ploeg L. H., (1996) A receptor in pituitary and hypothalamus that functions in growth hormone release. Science. 273, 974-977. DOI:10.1126/science.273.5277.974

13. Leung P. K., Chow K. B., Lau P. N., Chu K. M., Chan C. B., Cheng C. H. and Wise H., (2007) The truncated ghrelin receptor polypeptide (GHS-R1b) acts as a dominant-negative mutant of the ghrelin receptor. Cell. Signal. 19, 1011-1022. DOI:S0898-6568(06)00303-2

14. Navarro G., Aguinaga D., Angelats E., Medrano M., Moreno E., Mallol J., Cortes A., Canela E. I., Casado V., McCormick P. J., Lluis C. and Ferre S., (2016) A Significant Role of the Truncated Ghrelin Receptor GHS-R1b in Ghrelin-induced Signaling in Neurons. J. Biol. Chem. 291, 13048-13062. DOI:10.1074/jbc.M116.715144

15. Grosse R., Schoneberg T., Schultz G. and Gudermann T., (1997) Inhibition of gonadotropin-releasing hormone receptor signaling by expression of a splice variant of the human receptor. Mol. Endocrinol. 11, 1305-1318. DOI:10.1210/mend.11.9.9966

16. Osuga Y., Hayashi M., Kudo M., Conti M., Kobilka B. and Hsueh A. J., (1997) Co-expression of defective luteinizing hormone receptor fragments partially reconstitutes ligand-induced signal generation. J. Biol. Chem. 272, 25006-25012. doi: 10.1074/jbc.272.40.25006

17. Marklund U., Bystrom M., Gedda K., Larefalk A., Juneblad K., Nystrom S. and Ekstrand A. J., (2002) Intron-mediated expression of the human neuropeptide Y Y1 receptor. Mol. Cell. Endocrinol. 188, 85-97. DOI:S0303720701007389

18. Perron A., Sarret P., Gendron L., Stroh T. and Beaudet A., (2005) Identification and functional characterization of a 5-transmembrane domain variant isoform of the NTS2 neurotensin receptor in rat central nervous system. J. Biol. Chem. 280, 10219-10227. DOI:M410557200

19. Duran-Prado M., Gahete M. D., Martinez-Fuentes A. J., Luque R. M., Quintero A., Webb S. M., Benito-Lopez P., Leal A., Schulz S., Gracia-Navarro F., Malagon M. M. and Castano J. P., (2009) Identification and characterization of two novel truncated but functional isoforms of the somatostatin receptor subtype 5 differentially present in pituitary tumors. J. Clin. Endocrinol. Metab. 94, 2634-2643. DOI:10.1210/jc.2008-2564 [doi].
